# Feasibility of retrieval-augmented generation for large language models with Japanese input in radiotherapy

**DOI:** 10.1093/jrr/rrag019

**Published:** 2026-05-09

**Authors:** Yoshiyuki Takahashi, Noriyuki Kadoya, Kazuhiro Arai, Hikaru Tanno, Shohei Tanaka, Yoshiyuki Katsuta, Taichi Hoshino, Hinako Harada, So Omata, Takaya Yamamoto, Rei Umezawa, Keisuke Yasui, Naoki Hayashi, Keiichi Jingu

**Affiliations:** Department of Radiation Oncology, Tohoku University Graduate School of Medicine, 1-1 Seiryo-machi, Aoba-ku, Sendai, Miyagi 980-8574, Japan; Department of Radiation Oncology, Tohoku University Graduate School of Medicine, 1-1 Seiryo-machi, Aoba-ku, Sendai, Miyagi 980-8574, Japan; Department of Radiation Oncology, Tohoku University Graduate School of Medicine, 1-1 Seiryo-machi, Aoba-ku, Sendai, Miyagi 980-8574, Japan; Department of Radiation Oncology, Tohoku University Graduate School of Medicine, 1-1 Seiryo-machi, Aoba-ku, Sendai, Miyagi 980-8574, Japan; Department of Radiation Oncology, Tohoku University Graduate School of Medicine, 1-1 Seiryo-machi, Aoba-ku, Sendai, Miyagi 980-8574, Japan; Department of Radiation Oncology, Tohoku University Graduate School of Medicine, 1-1 Seiryo-machi, Aoba-ku, Sendai, Miyagi 980-8574, Japan; Department of Radiation Oncology, Tohoku University Graduate School of Medicine, 1-1 Seiryo-machi, Aoba-ku, Sendai, Miyagi 980-8574, Japan; Department of Radiation Oncology, Tohoku University Graduate School of Medicine, 1-1 Seiryo-machi, Aoba-ku, Sendai, Miyagi 980-8574, Japan; Department of Radiation Oncology, Tohoku University Graduate School of Medicine, 1-1 Seiryo-machi, Aoba-ku, Sendai, Miyagi 980-8574, Japan; Department of Radiation Oncology, Tohoku University Graduate School of Medicine, 1-1 Seiryo-machi, Aoba-ku, Sendai, Miyagi 980-8574, Japan; Department of Radiation Oncology, Tohoku University Graduate School of Medicine, 1-1 Seiryo-machi, Aoba-ku, Sendai, Miyagi 980-8574, Japan; Division of Medical Physics, School of Medical Sciences, Fujita Health University, 1-98, Dengakugakubo, Kutsukake, Toyoake, Aichi 470-1192, Japan; Division of Medical Physics, School of Medical Sciences, Fujita Health University, 1-98, Dengakugakubo, Kutsukake, Toyoake, Aichi 470-1192, Japan; Department of Radiation Oncology, Tohoku University Graduate School of Medicine, 1-1 Seiryo-machi, Aoba-ku, Sendai, Miyagi 980-8574, Japan

**Keywords:** radiotherapy, large language model, retrieval-augmented generation, artificial intelligence, medical physicist, radiation oncologist

## Abstract

Large language models (LLMs) have recently gained attention for their potential. However, concerns remain regarding their reliability due to limitations such as hallucinations and insufficient domain-specific knowledge. Retrieval-augmented generation (RAG) has emerged as a promising approach, enabling LLMs to reference external knowledge sources and generate accurate outputs. We aimed to clarify the potential of RAG-enhanced LLMs with Japanese input in the field of radiotherapy. This was assessed by evaluating performance on three certification examinations in Japan: the Japanese Medical Physicist Examination, the Japanese Board Examination for Radiologists, and the Japanese Board Examination for Radiation Oncologists. In this study, we constructed a RAG system named Rad-Hub, consisting of a Japanese Radiotherapy Knowledge Database (JRKD) and a retrieval framework built on Microsoft Azure. The JRKD was populated with 32 Japanese radiotherapy textbooks and clinical guidelines. We assessed its utility by inputting all multiple-choice questions from the three examinations into ChatGPT-4o, both with and without Rad-Hub, and recording the answers**.** They were then compared with reference answers determined by experienced medical physicists and radiation oncologists. Rad-Hub improved accuracy across all examinations. Accuracy increased from 77.0% ± 2.6% to 84.6% ± 1.5% in the Medical Physicist examination, from 74.9% ± 2.0% to 82.1% ± 1.1% in the Radiologist examination, and from 55.6% ± 4.4% to 71.7% ± 4.5% in the Radiation Oncologist examination. Performance gains ranged from 7.2% to 16.1%. These findings highlight the potential of RAG-enhanced LLMs, particularly ChatGPT-4o with Rad-Hub, for integration into radiotherapy applications, such as educational and clinical decision assistance.

## INTRODUCTION

Large language models (LLMs) are artificial intelligence systems designed to process natural language and generate responses that resemble human communication [1]. Their ability to process complex information has drawn substantial attention, particularly for supporting knowledge-intensive tasks across various fields, including health care [2]. In the radiation therapy field, several studies have recently explored the potential of LLMs for clinical decision support, professional training, and communication with patients [3–8]. However, despite their promise, their reliability remains a concern because their outputs do not always align with clinical expectations or established medical knowledge. Because LLMs draw on vast and diverse information sources during generation, they may occasionally produce statements that are outdated, unreliable, or insufficiently grounded in evidence. This phenomenon, known as LLM hallucination, can produce information that appears plausible but factually incorrect, making it difficult to rely on these models in clinical practice [9, 10].

Retrieval-Augmented Generation (RAG) offers a promising direction for addressing hallucinations in LLMs [11, 12]. Within a RAG architecture, the model supplements its internal knowledge by retrieving relevant information from curated external sources, such as specialized textbooks and clinical guidelines. Integrating these materials into the generation process strengthens the grounding of model outputs, improving answer accuracy and reducing errors arising from insufficient domain knowledge. Recent studies have demonstrated the potential of RAG-based systems in various medical domains, including radiology and gastroenterology [13, 14].

In the field of radiotherapy, RAG-based systems such as RTPhy-ChatBot have recently been developed to generate guideline-based responses using AAPM reports [15]. However, no study has evaluated the performance of RAG-based LLMs using Japanese radiotherapy resources. Previous studies have shown that LLM performance differs between English and Japanese. Etxaniz et al. reported that these models may not fully leverage their capabilities when prompted in non-English languages [16]. Harigai et al. reported that GPT-4 tends to show higher accuracy for English-translated questions compared with original Japanese questions in radiology [17]. Similar language-dependent gaps have also been observed for ChatGPT-4o in multilingual benchmark evaluations [18]. In addition, Chirkova et al. demonstrated that RAG systems themselves exhibit language-dependent variation, with notably more stable performance in English than in non-English languages [19]. Therefore, when applying RAG-based systems in Japan, it is essential to evaluate how accurately they respond to Japanese prompts and handle radiotherapy-specific domain knowledge using Japanese-language resources.

In this study, we present Rad-Hub, a RAG-based system consisting of a Japanese Radiotherapy Knowledge Database (JRKD) and a retrieval framework, both constructed on the Microsoft Azure platform (https://azure.microsoft.com). The JRKD is composed of 32 Japanese radiotherapy textbooks and clinical guidelines. Rad-Hub functions as an external knowledge system that supplies the LLM with relevant information retrieved from the JRKD. This structure enables the model to generate responses grounded in authoritative Japanese radiotherapy sources. We used ChatGPT-4o (OpenAI) as the underlying LLM model. We evaluated performance using multiple-choice questions (MCQs) from the Japanese Medical Physicist Board Examination, the Japanese Board Examination for Radiologists, and the Japanese Board Examination for Radiation Oncologists. The accuracy of ChatGPT-4o with and without Rad-Hub was compared to assess the impact of using RAG. We aimed to clarify the potential of RAG to enhance the performance of LLMs with Japanese input in the field of radiotherapy.

## MATERIALS AND METHODS

### RAG system architecture

**Fig. 1:**
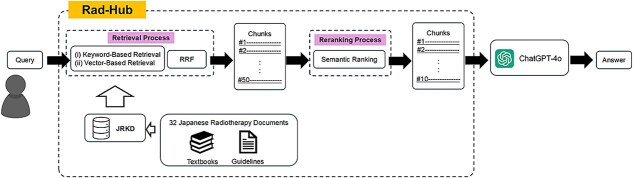
The overall workflow of Rad-Hub The process begins with converting a user query into a vector representation. The system then retrieves relevant segments from the JRKD through a hybrid process combining vector-based retrieval (HNSW) and keyword-based retrieval (BM25). The ranking results from these methods are then combined using Reciprocal Rank Fusion (RRF) to generate an integrated top-k candidate list. Next, the retrieved candidates are reordered through semantic ranking to prioritize the most relevant segments. Finally, the top-ranked segments are combined with the original query and passed to ChatGPT-4o to generate a grounded response.

The overall workflow of Rad-Hub is shown in Fig. 1. Rad-Hub was designed as a RAG system that integrates the JRKD with a retrieval framework, both constructed on the Microsoft Azure platform. The retrieval framework was implemented using Microsoft Azure AI Search. The system performs hybrid retrieval by combining keyword-based retrieval and vector-based retrieval, and applies semantic ranking as a subsequent step. According to the Azure AI Search documentation, hybrid retrieval with a semantic ranker offers significant benefits in search relevance across real-world and benchmark datasets [20]. Motivated by this evidence, Rad-Hub adopts the same architecture to ensure robust retrieval performance for highly technical Japanese radiotherapy terminology.

When a user enters a query, the system converts the input into a vector representation using the text-embedding-ada-002 model provided through the Azure OpenAI API. Azure AI Search then performs hybrid retrieval by applying keyword-based retrieval and vector-based retrieval to identify candidate text segments from the JRKD. After this initial retrieval, semantic ranking is applied to reorder the candidates based on contextual relevance. The top-ranked segments are subsequently provided to the downstream LLM, allowing it to generate responses grounded in Japanese radiotherapy knowledge**.** To enable this end-to-end pipeline, we implemented the integration and configuration by orchestrating the Azure OpenAI API and Azure AI Search.

### Construction of the JRKD

The JRKD was constructed using 32 Japanese radiotherapy documents, including textbooks and clinical guidelines, selected through expert consultation. To identify educational resources and clinical reference materials commonly used in routine practice, we interviewed six medical physicists and three radiation oncologists. The selected documents included the major Medical Physics Textbook Series—covering radiation physics, dosimetry, imaging and informatics, radiation therapy physics, diagnostic physics, nuclear medicine physics, and radiation protection—as well as widely used Japanese clinical textbooks. In addition, national Japanese clinical guidelines—such as those for lung, head and neck, liver, and pancreatic cancers—were incorporated. These documents were obtained in PDF format, either digitally or through scanning. When necessary, optical character recognition (OCR) was applied using an OCR engine (Adobe-based) to extract machine-readable text. The extracted text was manually reviewed during preprocessing to ensure readability and to minimize obvious OCR-related errors in highly technical radiotherapy content. A list of the 32 documents included in the JRKD is provided in the Supplementary Data.

To prepare the documents for retrieval process, the text was segmented into smaller chunks. We independently set the chunk size to a maximum of 500 tokens. Each chunk contained up to 500 tokens, a size chosen to balance semantic coherence with retrieval efficiency while minimizing context fragmentation in highly technical radiotherapy content. Each chunk was then converted into a vector representation using the same embedding model that was used to generate embeddings for user queries.

### L‌LM integration and RAG system workflow

In this study, we employed ChatGPT-4omni (ChatGPT-4o, OpenAI Inc., San Francisco, CA, USA) as the underlying LLM. ChatGPT, developed by OpenAI and first released in 2022, can generate fluent, human-like text in multiple languages, including Japanese. ChatGPT-4o, the most recent model available at the time of this study, was released in May 2024. Although recent studies have shown that ChatGPT-4o performs well on the Japanese Medical Licensing Examination and in clinical decision-making tasks in radiation oncology, hallucinations in specialized domains have also been reported [21, 22]. Therefore, we selected ChatGPT-4o as the underlying LLM while incorporating a RAG framework to improve the factual grounding of its responses.

The workflow of Rad-Hub combined with ChatGPT-4o proceeds through the following steps:

1. Query embedding process:

The user’s query is embedded using the same embedding model (*text-embedding-ada-002*) employed for generating vector representations of JRKD segments.

2. Retrieval process:

The system performs both vector-based and keyword-based retrieval. Vector similarity is computed using Hierarchical Navigable Small World (HNSW). Keyword-based retrieval is performed using BM25. The ranking results from these methods are then combined using Reciprocal Rank Fusion (RRF) to generate an integrated top-k candidate list.

3. Reranking process (semantic ranking):

The Semantic Ranker provided by Azure AI Search is applied to the top-k candidates obtained through RRF. This re-ranking process evaluates the contextual relevance between the user query and each candidate passage using a transformer-based semantic model and reorders the candidates to prioritize the most semantically relevant segments.

4. Response generation process:

The top-ranked segments after semantic ranking are combined with the original query and formatted into a prompt, which is then provided to ChatGPT-4o to generate a grounded response.

This workflow enables ChatGPT-4o to generate more accurate and well-grounded responses by leveraging the domain-specific information supplied through Rad-Hub. In addition, the JRKD can be updated efficiently, as newly released textbooks or clinical guidelines can be incorporated without retraining the model.

### Evaluation

**Fig. 2:**
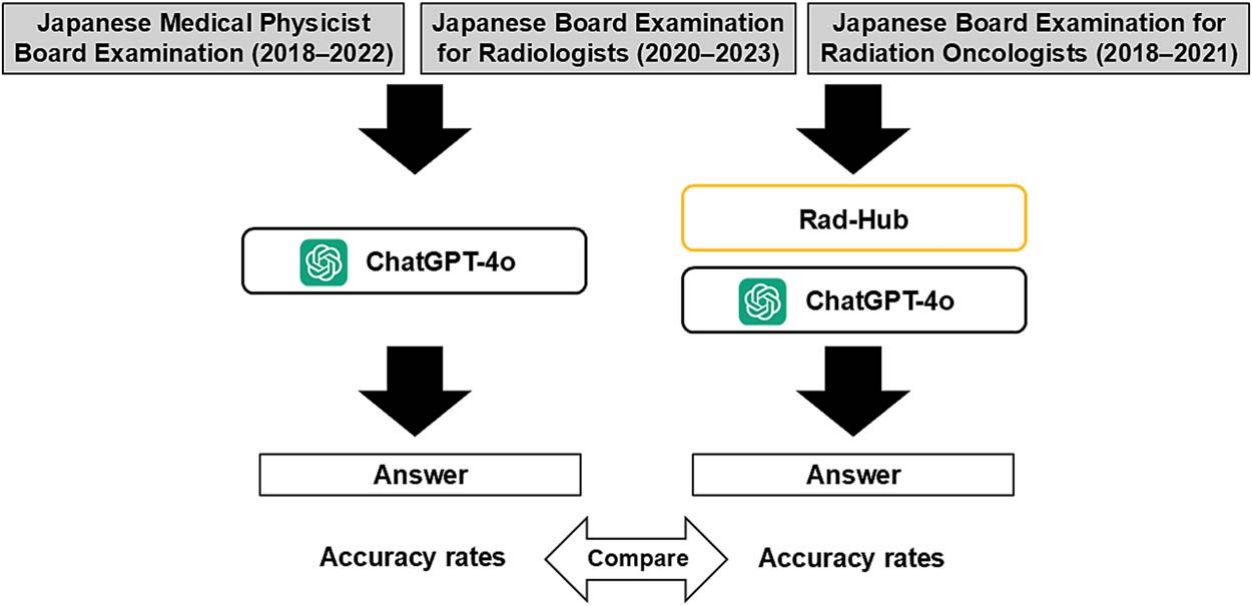
The overall workflow of the evaluation. MCQs from three radiotherapy-related professional board examinations administered in Japan—the Japanese Medical Physicist Board Examination (2018–22), the Japanese Board Examination for Radiologists (2020–23), and the Japanese Board Examination for Radiation Oncologists (2018–21)—were input into ChatGPT-4o with and without Rad-Hub, leveraging the JRKD. The generated answers were assessed using expert-determined correct answers, and the accuracy of the two models was compared.

An overview of the evaluation workflow is shown in Fig. 2.

We utilized questions from three radiotherapy-related professional board examinations administered in Japan: the Japanese Medical Physicist Board Examination (2018–22), the Japanese Board Examination for Radiologists (2020–23), and the Japanese Board Examination for Radiation Oncologists (2018–21). These examinations cover a range of question formats, including MCQs. In the Japanese medical system, medical physicists specializing in radiation therapy are required to pass the Medical Physicist Board Examination, whereas radiation oncologists first pass the Radiologist Board Examination before pursuing certification through the Radiation Oncologist Board Examination. Because these examinations assess the essential baseline knowledge required in radiotherapy practice, we considered them suitable benchmarks for evaluating fundamental Japanese radiotherapy knowledge. In addition, the questions are standardized, expert-validated assessments written in Japanese, making them particularly appropriate for evaluating whether an LLM can correctly process domain-specific Japanese medical information. Therefore, we used these examinations to evaluate the performance of ChatGPT-4o, with and without Rad-Hub, leveraging the JRKD.

For this study, we focused exclusively on MCQs to evaluate the text-based reasoning capabilities of LLMs. We excluded questions that required materials that could not be directly input into the models, such as visual materials (e.g. radiographic or CT images) and questions whose correct answers were affected by updates in clinical guidelines. In addition, expert reviewers excluded questions that were considered inappropriate or ambiguous, including those that did not allow a uniquely correct answer or for which multiple answer choices could reasonably be considered correct. Exclusion criteria were categorized as follows: (i) conflicts with updated clinical guidelines, (ii) items requiring materials that could not be directly input into the models, and (iii) items determined to be inappropriate by expert evaluators. From the medical physicist examination, the numbers of excluded questions were 5 in 2018, 6 in 2019, 5 in 2020, 12 in 2021, and 13 in 2022. From the radiologist examination, the numbers of excluded questions were 4 in 2020, 0 in 2021, 4 in 2022, and 1 in 2023. From the radiation oncologist examination, the numbers of excluded questions were 5 in 2018, 3 in 2019, 5 in 2020, and 8 in 2021. After applying these exclusion criteria, a total of 709 questions from the medical physicist examination, 391 questions from the radiologist examination, and 232 questions from the radiation oncologist examination were used in this study. For the Medical Physicist examination, the excluded question numbers were 8, 9, 26, 30, and 59 (Medicine/Biology) and 5, 16, 51, 52, 71, 73, 74, and 80 (Physics/Engineering) in 2022; 2, 11, 16, 22, and 54 (Medicine/Biology) and 16, 25, 50, 53, 57, 61, and 79 (Physics/Engineering) in 2021; 54, 55, 64, 68, and 73 (Physics/Engineering) in 2020; 1 (Medicine/Biology) and 21, 48, 54, 56, and 61 (Physics/Engineering) in 2019; and 56 (Medicine/Biology) and 14, 56, 59, and 62 (Physics/Engineering) in 2018. (For the Japanese radiologist and radiation oncologist examinations, the number of excluded questions was not reported, as these examinations were not publicly available.)

All questions were entered into ChatGPT-4o both with and without Rad-Hub. After collecting all responses, the outputs generated by ChatGPT-4o with and without Rad-Hub were compared with the correct answers determined by experienced medical physicists and radiation oncologists (with over 6 years of clinical experience), as the official answers to these Japanese board examinations are not publicly available [8]. For statistical analysis, the McNemar test was used to determine if the accuracy rate in two groups significantly differs from each other. Differences with a *P* value of <0.05 were considered significant.

## RESULT

**Fig. 3:**
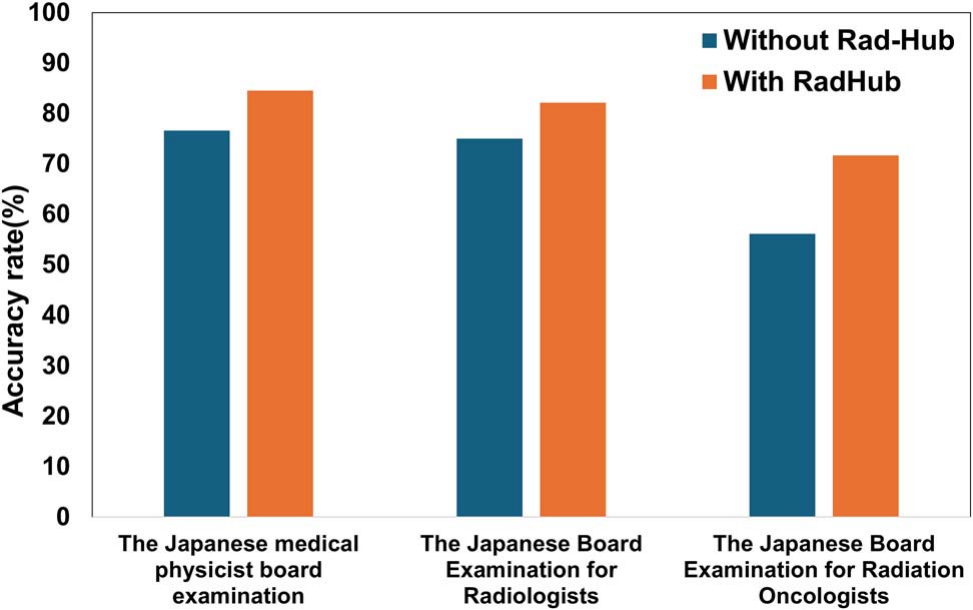
Summary of the overall accuracy rates of ChatGPT-4o with and without Rad-Hub. The orange bars represent accuracy rates with Rad-Hub, and the blue bars represent accuracy rates without Rad-Hub.

Fig. 3 summarizes the accuracy rates for each examination, and the detailed values are presented in Table 1. Overall accuracy, calculated across all categories, differed significantly between ChatGPT-4o with and without Rad-Hub across all three examinations (*P*-value <0.05). For the Japanese Medical Physicist Board Examination, the average accuracy rates were 77.0% ± 2.6% without Rad-Hub and 84.6% ± 1.5% with Rad-Hub. For the Japanese Board Examination for Radiologists, the corresponding accuracy rates were 74.9% ± 2.0% and 82.1% ± 1.1%. In the Japanese Board Examination for Radiation Oncologists, the average accuracy rates were 55.6% ± 4.4% without Rad-Hub and 71.7% ± 4.5% with Rad-Hub. Across all three examinations, Rad-Hub consistently improved accuracy, with performance gains ranging from 7.2% to 16.1%.

**Table 1:** Summary of accuracy rates with (w/) and without (w/o) Rad-Hub

**Medical physicist examination**	Accuracy rate (%)			Accuracy rate (%)			Accuracy rate (%)			Accuracy rate (%)			Accuracy rate (%)			Accuracy rate (%)		
	2018			2019			2020			2021			2022			Average		
category	Number of questions	w/o Rad-Hub	w/ Rad-Hub	Number of questions	w/o Rad-Hub	w/ Rad-Hub	Number of questions	w/o Rad-Hub	w/ Rad-Hub	Number of questions	w/o Rad-Hub	w/ Rad-Hub	Number of questions	w/o Rad-Hub	w/ Rad-Hub	Number of questions	w/o Rad-Hub	w/ Rad-Hub
basic medicine	20	95.0	95.0	19	84.2	94.7	20	85.0	95.0	17	94.1	94.1	18	88.9	94.4	94	89.4 ± 4.5	94.7 ± 0.3
diagnostic radiology	10	80.0	100.0	10	100.0	100.0	10	90.0	100.0	9	77.8	88.9	8	100.0	100.0	47	89.6 ± 9.5	97.8 ± 4.4
nuclear medicine	10	90.0	90.0	10	80.0	80.0	10	90.0	100.0	10	80.0	80.0	10	90.0	90.0	50	86.0 ± 4.9	88.0 ± 7.5
radiation oncology	10	60.0	80.0	10	100.0	100.0	10	90.0	100.0	10	90.0	80.0	10	60.0	60.0	50	80.0 ± 16.7	84.0 ± 15.0
radiation biology	9	55.6	77.8	10	60.0	80.0	10	70.0	80.0	9	88.9	88.9	9	88.9	88.9	47	72.7 ± 14.0	83.1 ± 4.8
radiation physics	14	92.9	92.9	15	80.0	86.7	15	73.3	73.3	15	86.7	86.7	14	78.6	92.9	73	82.3 ± 6.8	86.5 ± 7.1
statistics	5	100.0	100.0	5	100.0	100.0	5	100.0	100.0	4	100.0	100.0	4	100.0	100.0	23	100.0 ± 0.0	100.0 ± 0.0
health physics/radiological protection	10	80.0	80.0	9	77.8	88.9	10	80.0	90.0	9	55.6	88.9	10	50.0	60.0	48	68.7 ± 13.1	81.6 ± 11.4
diagnostic radiation physics	10	60.0	80.0	10	50.0	70.0	10	80.0	80.0	10	40.0	50.0	10	90.0	100.0	50	64.0 ± 18.5	76.0 ± 16.2
nuclear medicine physics	10	70.0	80.0	9	66.7	77.8	10	50.0	70.0	9	44.4	55.6	10	80.0	80.0	48	62.2 ± 13.1	72.7 ± 9.3
radiation therapy physics	8	62.5	62.5	8	50.0	75.0	8	87.5	100.0	8	87.5	87.5	8	62.5	62.5	40	70.0 ± 15.0	77.5 ± 14.6
radiation metrology	9	55.6	66.7	9	66.7	88.9	8	87.5	87.5	9	77.8	66.7	10	50.0	60.0	45	67.5 ± 13.8	73.9 ± 11.9
medical and imaging informatics	10	90.0	90.0	10	70.0	90.0	9	77.8	77.8	9	88.9	100.0	6	100.0	100.0	44	85.3 ± 10.4	91.6 ± 8.2
radiation-related laws and recommendations/medical ethics	10	40.0	70.0	10	30.0	60.0	10	60.0	70.0	10	60.0	80.0	10	80.0	90.0	50	54.0 ± 17.4	74.0 ± 10.2
Total (all categories)	145	75.2	84.1	144	72.9	85.4	145	79.3	86.9	138	79.0	82.6	137	78.8	83.9	709	77.0 ± 2.6	84.6 ± 1.5
**Radiologist examination**	Accuracy rate (%)			Accuracy rate (%)			Accuracy rate (%)			Accuracy rate (%)			Accuracy rate (%)					
	2020			2021			2022			2023			Average					
category	Number of questions	w/o Rad-Hub	w/ Rad-Hub	Number of questions	w/o Rad-Hub	w/ Rad-Hub	Number of questions	w/o Rad-Hub	w/ Rad-Hub	Number of questions	w/o Rad-Hub	w/ Rad-Hub	Number of questions	w/o Rad-Hub	w/ Rad-Hub			
diagnostic radiology	41	70.7	75.6	52	71.2	80.8	52	76.9	76.9	50	72.0	74.0	195	72.7 ± 2.5	76.8 ± 2.5			
interventional radiology	2	50.0	50.0	3	66.7	100.0	3	33.3	66.7	3	0.0	33.3	11	37.5 ± 24.7	62.5 ± 24.7			
nuclear medicine	10	80.0	90.0	15	93.3	93.3	15	86.7	93.3	15	100.0	100.0	55	90.0 ± 7.5	94.2 ± 3.6			
radiation oncology	17	94.1	94.1	22	81.8	90.9	22	54.5	77.3	22	77.3	90.9	83	76.9 ± 14.3	88.3 ± 6.5			
general radiological knowledge	11	63.6	90.9	13	53.8	61.5	9	77.8	88.9	14	92.9	92.9	47	72.0 ± 14.7	83.5 ± 12.8			
Total (all categories)	81	75.3	82.7	105	74.3	82.9	101	72.3	80.2	104	77.9	82.7	391	74.9 ± 2.0	82.1 ± 1.1			
**Radiation oncologist examination**	Accuracy rate (%)			Accuracy rate (%)			Accuracy rate (%)			Accuracy rate (%)			Accuracy rate (%)					
	2018			2019			2020			2021			Average					
	Number of questions	w/o Rad-Hub	w/ Rad-Hub	Number of questions	w/o Rad-Hub	w/ Rad-Hub	Number of questions	w/o Rad-Hub	w/ Rad-Hub	Number of questions	w/o Rad-Hub	w/ Rad-Hub	Number of questions	w/o Rad-Hub	w/ Rad-Hub			
Total (all categories)	61	59.0	73.8	63	49.2	73.0	50	54.0	64.0	58	60.3	75.9	232	55.6 ± 4.4	71.7 ± 4.5			

The average time from question input to completion of answer generation was approximately 15 seconds with Rad-Hub and approximately 3 seconds without Rad-Hub.

**Fig. 4:**
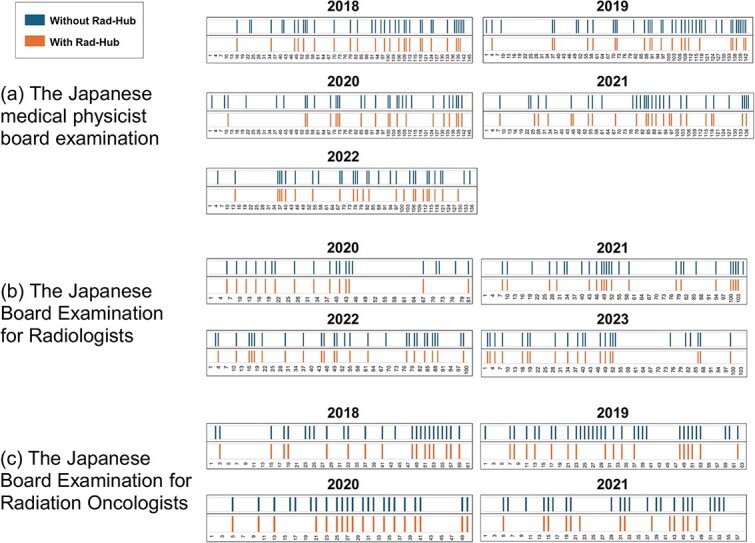
Distribution of incorrectly answered questions with and without Rad-Hub across the three examinations: (a) the Japanese Medical Physicist Examination, (b) the Japanese Board Examination for Radiologists, and (c) the Japanese Board Examination for Radiation Oncologists. The orange bars indicate results with Rad-Hub, and the blue bars indicate results without Rad-Hub.

Fig. 4 illustrates the distribution of incorrectly answered questions with and without Rad-Hub across the three examinations. Rad-Hub corrected many errors made without it, although it also introduced a small number of new errors, as shown in Fig. 4.

**Fig. 5:**
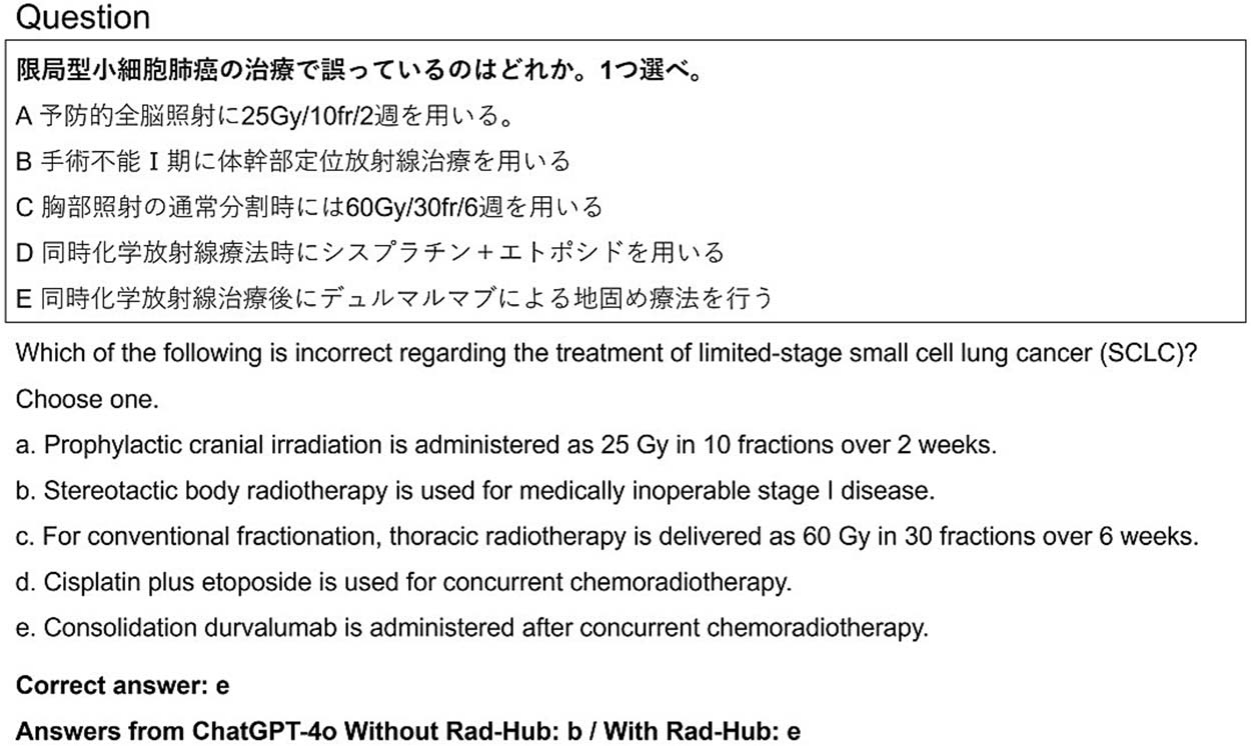
Example question in the case that Rad-Hub corrected an error made without it (Question 23 in the Radiation Oncologists Examination 2020).

**Fig. 6:**
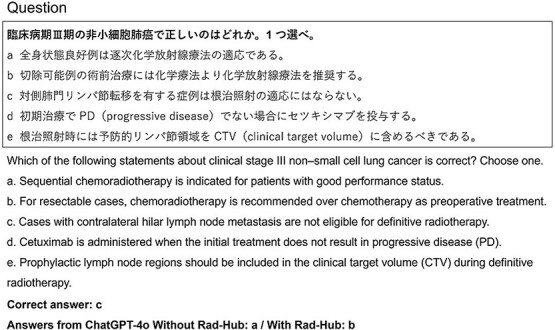
Example question in the case that ChatGPT-4o answered incorrectly both with and without Rad-Hub (Question 78 in the Radiologist Examination 2021).

**Fig. 7:**
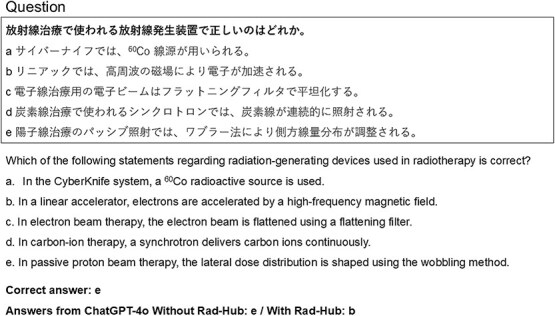
Example question in the case that ChatGPT-4o answered incorrectly with Rad-Hub, while correctly answered without it (Question 60 in the Medical Physicist Examination 2022 physics and engineering).

To provide a qualitative perspective, Figs 5–7 present representative cases illustrating how Rad-Hub influenced model responses. Fig. 5 shows an example in which a question answered incorrectly without Rad-Hub was answered correctly when Rad-Hub was used. In contrast, Fig. 6 shows a case in which the correct answer could not be identified with or without Rad-Hub. Fig. 7 illustrates a contrasting case in which Rad-Hub led to an incorrect answer, whereas ChatGPT-4o, without Rad-Hub, answered correctly.

## DISCUSSION

In this study, we evaluated the performance of ChatGPT-4o with and without Rad-Hub on three Japanese board examinations related to radiotherapy: the Medical Physicist, Radiologist, and Radiation Oncologist examinations. Notably, Rad-Hub improved accuracy by up to 16.1% and consistently enhanced performance across all examinations, demonstrating its potential to strengthen domain-specific knowledge in Japanese-language radiotherapy contexts. These findings highlight the importance of incorporating structured, domain-relevant knowledge sources when applying LLMs to highly specialized medical tasks.

Our findings are consistent with recent work by Thaker et al., who evaluated 15 LLMs—including ChatGPT-4o—using 298 scorable questions from the 2021 American College of Radiology in-training examination [23]. In their study, RadOncRAG augmented user prompts with information retrieved from a radiation oncology domain knowledge database comprising textbooks, protocols, clinical guidelines, and question-and-answer banks. Notably, ChatGPT-4o demonstrated a marked improvement in accuracy when supported by this RAG framework, increasing from 79.9% to 85.6%. These results closely align with our observations that incorporating domain-specific Japanese radiotherapy knowledge through Rad-Hub substantially enhances ChatGPT-4o’s performance. Importantly, our study extends this line of evidence to the Japanese clinical context and is the first to demonstrate improved accuracy across the Japanese Medical Physicist, Radiologist, and Radiation Oncologist board examinations, reinforcing the feasibility of deploying RAG-enhanced LLMs in Japanese-language radiotherapy education and clinical support.

The improvement in accuracy observed with Rad-Hub is explained by the system’s design to retrieve and reference appropriate radiotherapy knowledge from the JRKD at the time of answering. The JRKD consists of Japanese-language textbooks and clinical guidelines selected by experts and reflects standard radiotherapy knowledge in Japan. Rad-Hub retrieves relevant passages from these curated resources and incorporates them as segmented Japanese text during response generation, allowing ChatGPT-4o to use additional domain information at inference time. In addition, presenting retrieved evidence in Japanese may improve alignment between the model’s responses and the wording and terminology used in Japanese professional examinations. Relatedly, cross-lingual RAG has highlighted that mismatches between the language of a query and that of retrieved evidence can affect performance and increase the difficulty of evidence-grounded reasoning [24]. Accordingly, providing JRKD information directly as Japanese text segments was intended to support both domain knowledge supplementation and alignment with Japanese radiotherapy phrasing. This design enables the model to correctly answer questions that ChatGPT-4o fails to answer.

In many questions for which the JRKD provided guideline support, Rad-Hub enabled ChatGPT-4o to correct errors that occurred without it. Fig. 5 illustrates one such case. The question required guideline-specific knowledge regarding limited-stage small cell lung cancer (SCLC). Without Rad-Hub, ChatGPT-4o selected option B, incorrectly judging stereotactic body radiotherapy for medically inoperable stage I disease as inappropriate. In contrast, with Rad-Hub, the model correctly selected option E, recognizing that consolidation durvalumab after concurrent chemoradiotherapy—although recommended for unresectable stage III non–small cell lung cancer (NSCLC)—is not indicated for limited-stage SCLC according to the 2022 Japanese lung cancer guidelines included in the JRKD. This example demonstrates how Rad-Hub supplies targeted, guideline-level information that helps ChatGPT-4o generate answers consistent with disease-specific recommendations. It highlights a key advantage of radiotherapy-focused RAG systems: the ability to correct guideline-inconsistent answers by retrieving precise and clinically relevant guidance at inference time.

Although Rad-Hub accurately answered most questions for which the JRKD contained explicit supporting information, a small number of questions remained incorrect despite the presence of relevant guideline content. Fig. 6 illustrates one such example. Option b states that preoperative chemoradiotherapy is recommended for all resectable cases, implying applicability across the entire stage III population. In contrast, the 2022 Japanese Lung Cancer Guidelines specify that preoperative chemoradiotherapy may be considered only in selected stage IIIA cases. Meanwhile, option c addresses contralateral hilar lymph node metastasis, which the guidelines explicitly identify as not being an indication for curative radiotherapy. From a clinical standpoint, option c is clearly the appropriate choice according to the guideline recommendations contained within the JRKD. ChatGPT-4o—with or without Rad-Hub—was unable to integrate these distinctions and therefore failed to reach the correct conclusion. A possible technical factor is the ranking of retrieved guideline passages within Rad-Hub. Because “stage III” and “stage IIIA” have highly similar vector representations, the retrieval pipeline may have prioritized text related to preoperative chemoradiotherapy (option b) over the clearer guidance on contralateral hilar lymph node involvement (option c). This imbalance may have contributed to the model’s incorrect conclusion.

In contrast, Fig. 7 presents an example in which the application of Rad-Hub led to an incorrect answer, whereas ChatGPT-4o without Rad-Hub correctly selected the appropriate option. The question assessed fundamental knowledge of radiation-generating devices used in radiotherapy, for which option (e) was the correct answer. Without Rad-Hub, ChatGPT-4o correctly identified option (e), which states that in passive proton beam therapy, the lateral dose distribution is shaped using the wobbling method. However, when Rad-Hub was applied, the model instead selected option (b), incorrectly indicating that electrons in a medical linear accelerator are accelerated by a high-frequency magnetic field. This statement is incorrect because electrons in a medical linear accelerator are accelerated by a high-frequency electric field, not a magnetic field. One possible explanation for this behavior is that the additional context provided by Rad-Hub influenced the model’s final decision, even though the model originally possessed the correct knowledge. In this case, option (b), which includes technical terms such as “high-frequency,” “magnetic field,” and “particle acceleration,” may have appeared more convincing when interpreted together with the retrieved domain-specific context, despite being incorrect. In particular, because electric and magnetic fields are often discussed together in descriptions of accelerator systems, the retrieved context may have reduced the clarity of their distinct roles, effectively overriding the model’s original correct reasoning. Prior work has reported that in RAG, retrieved context that is semantically related but does not contain the answer can distract the model’s judgment and lead to incorrect answers [25]. To address this limitation, recent work has explored mechanisms for explicitly verifying the consistency between retrieved information and the model’s internal knowledge, aiming to reduce errors caused by over-reliance on external context [26]. These findings suggest that, for radiotherapy-related applications, further efforts to improve the accuracy and reliability of RAG-based systems may be worth considering.

This study has several limitations. First, our evaluation was limited to text-based board examination questions, and we did not assess the model’s performance on image-based items, which constitute an important component of radiotherapy practice. Recent advances in hybrid RAG-based multimodal LLMs have demonstrated the potential of combining text and imaging features to improve performance in medical tasks [27]. Incorporating multimodal retrieval and inference may therefore further improve the robustness of Japanese-language radiotherapy LLMs. Second, this study assessed LLM performance exclusively using board examination questions rather than real clinical cases. Although board examinations provide a standardized method for evaluating domain knowledge, they do not fully reflect the complexity of real-world radiotherapy practice, where clinical decision-making involves imaging interpretation, patient-specific factors, and multidisciplinary considerations. Future studies incorporating actual clinical scenarios will be necessary to determine the practical utility of Rad-Hub in routine practice. Finally, the proposed system was implemented on a cloud-based infrastructure using ChatGPT-4o and Rad-Hub. Recent systematization studies of RAG suggest that cloud-hosted and third-party components may increase privacy and information-leakage risks, which can limit direct deployment in clinical environments with strict information-security and data-governance requirements [28]. Local or on-premises implementations will be necessary for future clinical integration of radiotherapy RAG-based LLMs. Despite these limitations, this study provides a foundational step toward the development of Japanese-language RAG systems for radiotherapy.

## CONCLUSIONS

We evaluated the performance of ChatGPT-4o with and without Rad-Hub, a RAG system that references Japanese radiotherapy textbooks and clinical guidelines, using questions from three Japanese board examinations related to radiotherapy. Across all examinations, Rad-Hub consistently improved accuracy, yielding gains of up to 16.1% and reducing errors attributable to insufficient grounding in domain-specific knowledge. By directly comparing accuracy under the two conditions, this study demonstrates that using a RAG system such as Rad-Hub can enhance ChatGPT-4o’s performance on Japanese radiotherapy examination questions. Although these findings are limited to examination settings, the observed improvements suggest that RAG-based approaches have the potential to strengthen LLM performance for Japanese input in radiotherapy contexts. These results indicate the feasibility of applying RAG-based LLMs to future radiotherapy applications such as educational support and guideline-concordant clinical decision assistance.

## Supplementary Material

Supplementary_Data_rrag019

## References

[1] Radford A, Narasimhan K, Salimans T et al. Improving language understanding by generative pre-training. OpenAI 2018.

[2] Thirunavukarasu AJ, Ting DSJ, Elangovan K et al. Large language models in medicine. Nat Med 2023;29:1930–40. 10.1038/s41591-023-02448-8. 37460753

[3] Kadoya N, Arai K, Tanaka S et al. Assessing knowledge about medical physics in language-generative AI with large language model: using the medical physicist exam. Radiol Phys Technol 2024;17:929–37. 10.1007/s12194-024-00838-2. 39254919

[4] Toyama Y, Harigai A, Abe M et al. Performance evaluation of ChatGPT, GPT-4, and bard on the official board examination of the Japan radiology society. Jpn J Radiol 2024;42:201–7. 10.1007/s11604-023-01491-2. 37792149 PMC10811006

[5] Putz F, Haderlein M, Lettmaier S et al. Exploring the capabilities and limitations of large language models for radiation oncology decision support. International Journal of Radiation Oncology*Biology*Physics 2024;118:900–4. 10.1016/j.ijrobp.2023.11.062. 38401978

[6] Dennstädt F, Hastings J, Putora PM et al. Exploring capabilities of large language models such as ChatGPT in radiation oncology. Advances in Radiation Oncology 2024;9:101400. 10.1016/j.adro.2023.101400. 38304112 PMC10831180

[7] Rebelo N, Sanders L, Li K et al. Learning the treatment process in radiotherapy using an artificial intelligence–assisted Chatbot: development study. JMIR Formative Research 2022;6:e39443. 10.2196/39443. 36327383 PMC9718518

[8] Kadoya N, Takahashi Y, Koga S et al. Evaluating the capability of large language models in radiotherapy through professional certification examinations in Japan. J Radiat Res 2026;67:114–20. 10.1093/jrr/rraf083. 41518145 PMC12856024

[9] Huang L, Yu W, Ma W et al. A survey on hallucination in large language models: principles, taxonomy, challenges, and open questions. ACM Trans Inf Syst 2025;43:1–42:55. 10.1145/3703155.

[10] Keshavarz P, Bagherieh S, Nabipoorashrafi SA et al. ChatGPT in radiology: a systematic review of performance, pitfalls, and future perspectives. Diagnostic and Interventional Imaging 2024;105:251–65. 10.1016/j.diii.2024.04.003. 38679540

[11] Lewis P, Perez E, Piktus A et al. Retrieval-augmented generation for knowledge-intensive NLP tasks. In: Advances in Neural Information Processing Systems, Vol. 33. Curran Associates, Inc, 2020, 9459–74.

[12] Shuster K, Poff S, Chen M et al. Retrieval augmentation reduces hallucination in conversation. In: Moens M-F, Huang X, Specia L et al. (eds). Findings of the Association for Computational Linguistics: EMNLP 2021. Punta Cana, Dominican Republic: Association for Computational Linguistics, 2021, 3784–803.

[13] Tozuka R, Johno H, Amakawa A et al. Application of NotebookLM, a large language model with retrieval-augmented generation, for lung cancer staging. Jpn J Radiol 2024;43:706–12. 10.1007/s11604-024-01705-1. 39585559

[14] Zhou Q, Liu C, Duan Y et al. GastroBot: a Chinese gastrointestinal disease chatbot based on the retrieval-augmented generation. Front Med (Lausanne) 2024;11:1392555. 10.3389/fmed.2024.1392555. 38841582 PMC11150590

[15] Wei S, Hu A, Wang Z et al. RTPhy-ChatBot: a RAG-based intelligent assistant for radiotherapy physics using LLaMA3 and AAPM reports. Journal of Applied Clinical Medical Physics 2025;26:e70263. 10.1002/acm2.70263. 41065312 PMC12509246

[16] Etxaniz J, Azkune G, Soroa A et al. Do multilingual language models think better in English? arXiv.org 2023, arXiv:2308.01223.

[17] Harigai A, Toyama Y, Nagano M et al. Response accuracy of GPT-4 across languages: insights from an expert-level diagnostic radiology examination in Japan. Jpn J Radiol 2025;43:319–29. 10.1007/s11604-024-01673-6. 39466356 PMC11790683

[18] Xuan W, Yang R, Qi H et al. MMLU-ProX: A multilingual benchmark for advanced large language model evaluation. In: Christodoulopoulos C, Chakraborty T, Rose C et al. (eds). Proceedings of the 2025 Conference on Empirical Methods in Natural Language Processing. Suzhou, China: Association for Computational Linguistics, 2025, 1513–32.

[19] Chirkova N, Rau D, Déjean H et al. Retrieval-augmented generation in multilingual settings. arXiv.org 2024, arXiv:2407.01463.

[20] HeidiSteen . Azure AI Search Documentation. URL: https://learn.microsoft.com/en-us/azure/search/ [accessed 2025–11–16].

[21] Miyazaki Y, Hata M, Omori H et al. Performance of ChatGPT-4o on the Japanese medical licensing examination: evalution of accuracy in text-only and image-based questions. JMIR Medical Education 2024;10:e63129. 10.2196/63129. 39718557 PMC11687171

[22] Chuang W-K, Kao Y-S, Liu Y-T et al. Assessing ChatGPT for clinical decision-making in radiation oncology, with open-ended questions and images. Pract Radiat Oncol 2025;15:e412–23. 10.1016/j.prro.2025.04.009. 40311921

[23] Thaker NG, Redjal N, Dicker A et al. RadOncRAG: a novel retrieval-augmented generation framework improves large language model benchmark performance in radiation oncology. JCO Clin Cancer Inform 2025;9:e2500220. 10.1200/CCI-25-00220. 41237352

[24] Liu W, Trenous S, Ribeiro LFR et al. XRAG: cross-lingual retrieval-augmented generation. In: Christodoulopoulos C, Chakraborty T, Rose C et al. (eds). Findings of the Association for Computational Linguistics: EMNLP 2025. Suzhou, China: Association for Computational Linguistics, 2025, 15669–90.

[25] Cuconasu F, Trappolini G, Siciliano F et al. The power of noise: redefining retrieval for RAG systems. In: Proceedings of the 47th International ACM SIGIR Conference on Research and Development in Information Retrieval. New York, NY, USA: Association for Computing Machinery, 2024, 719–29.

[26] Zeng S, Zhang J, Li B et al. Towards knowledge checking in retrieval-augmented generation: a representation perspective. In: Chiruzzo L, Ritter A, Wang L (eds). Proceedings of the 2025 Conference of the Nations of the Americas Chapter of the Association for Computational Linguistics: Human Language Technologies (Volume 1: Long Papers). Albuquerque, New Mexico: Association for Computational Linguistics, 2025, 2952–69.

[27] Su C, Wen J, Kang J et al. Hybrid RAG-empowered multimodal LLM for secure data Management in Internet of medical things: a diffusion-based contract approach. IEEE Internet Things J 2025;12:13428–40. 10.1109/JIOT.2024.3521425.

[28] A-EBodea , SMeisenbacher, AKlymenko et al. SoK: Privacy Risks and Mitigations in Retrieval-Augmented Generation Systems. 2026, arXiv:2601.03979, 10.48550/arXiv.2601.03979.

