# Supplementary material for: Feasibility of retrieval-augmented generation for large language models with Japanese input in radiotherapy
Source: J Radiat Res. 2026 May 9;67(3):420–9. doi: 10.1093/jrr/rrag019 (PMC13202343; doi:10.1093/jrr/rrag019)
Supplement: Supplementary_Data_rrag019 [file supplementary_data_rrag019.docx]

Supplementary Data

| No | Author/Editor/Organization | Title (romanized [English]) | Publisher | Year (published) | URL (web only) |
| --- | --- | --- | --- | --- | --- |
| 1 | Sakae T, Endo M | Igaku Butsurigaku Kyokasho: Hoshasen Butsurigaku [Medical physics textbook: radiation physics] | Kokusai Bunken Sha | 2019 |  |
| 2 | Araki F | Igaku Butsurigaku Kyokasho: Hoshasen Chiryo Butsurigaku [Medical physics textbook: radiation therapy physics] | Kokusai Bunken Sha | 2016 |  |
| 3 | Matsumoto M | Igaku Butsurigaku Kyokasho: Hoshasen Shindan Butsurigaku [Medical physics textbook: diagnostic radiology physics] | Kokusai Bunken Sha | 2017 |  |
| 4 | Murayama H | Igaku Butsurigaku Kyokasho: Kakuigaku Butsurigaku [Medical physics textbook: nuclear medicine physics] | Kokusai Bunken Sha | 2015 |  |
| 5 | Akahane K, Endo M | Igaku Butsurigaku Kyokasho: Iryo Hoshasen Bogo-gaku [Medical physics textbook: medical radiation protection] | Kokusai Bunken Sha | 2020 |  |
| 6 | Nohtomi A | Igaku Butsurigaku Kyokasho: Hoshasen Keisokugaku [Medical physics textbook: radiation measurements] | Kokusai Bunken Sha | 2015 |  |
| 7 | Ogawa K | Igaku Butsurigaku Kyokasho: Gazo / Joho Shori [Medical physics textbook: image and information processing] | Kokusai Bunken Sha | 2018 |  |
| 8 | Japanese Society of Medical Physics | Gaibu hoshasen chiryo ni okeru mizu kyushu senryo no hyojun keisokuho: Hyojun keisokuho 12 [Standard dosimetry of absorbed dose to water in external beam radiotherapy: Standard dosimetry method 12] | Tsusho Sangyo Kenkyusha | 2023 |  |
| 9 | Onishi H, Karasawa K, Nishio T et al | Gan Hoshasen Ryoho. 8th ed. [Cancer and radiation therapy, 8th ed.] | Gakken | 2023 |  |
| 10 | Japanese Society of Medical Oncology | Shin rinsho shuyogaku. 7th ed. [New clinical oncology, 7th ed.] | Nankodo | 2024 |  |
| 11 | Sasaki T, Fujibuchi T, Atsumi K | Hoshasen chiryo gijutsugaku [Radiotherapy technology] | Nanzando | 2022 |  |
| 12 | Takeda A, Sanuki N, Oku Y | THE SBRT BOOK: Taikanbu teii hoshasen chiryo [THE SBRT BOOK: stereotactic body radiotherapy] | Shinohara Shuppan Shinsha | 2016 |  |
| 13 | Japanese Society for Radiation Oncology | Hoshasen chiryo keikaku guideline 2020-nenban [Radiation therapy planning guideline 2020] | Kanehara Shuppan | 2020 |  |
| 14 | Japanese Society for Radiation Oncology | Kanjashan to kazoku no tame no hoshasen chiryo Q&A 2020-nenban [Radiation therapy Q&A for patients and families 2020] | Kanehara Shuppan | 2020 |  |
| 15 | Japan Pancreas Society Pancreatic Cancer Guidelines Revision Committee | Suizo gan shinryo guideline 2022-nenban [Clinical practice guidelines for pancreatic cancer 2022] | Kanehara Shuppan | 2022 |  |
| 16 | Japan Lung Cancer Society | Haigan shinryo guideline 2022-nenban [Clinical practice guidelines for lung cancer 2022] | Kanehara Shuppan | 2022 |  |
| 17 | Japan Society for Head and Neck Cancer | Tokeibu gan shinryo guideline 2022-nenban [Clinical practice guidelines for head and neck cancer 2022] | Kanehara Shuppan | 2022 |  |
| 18 | Japan Society of Hepatology | Kangan shinryo guideline 2021-nenban [Clinical practice guidelines for hepatocellular carcinoma 2021] | Kanehara Shuppan | 2021 |  |
| 19 | Japan Society of Gynecologic Oncology | Shikyukeigan chiryo guideline 2022-nenban [Treatment guidelines for cervical cancer 2022] | Kanehara Shuppan | 2022 |  |
| 20 | Karasawa K, Narita Y, Ozawa S | Kyodo hencho hoshasen chiryo chiryo keikaku no kotsu [Tips for IMRT treatment planning] | Gakken | 2022 |  |
| 21 | Kurooka M, Miyaura K, Wakita A et al | Shosetsu hoshasen chiryo no seido kanri to sokutei gijutsu [Detailed QA and measurement techniques in radiotherapy] | Chugai Igakusha | 2012 |  |
| 22 | Okumura M, Kumazaki Y, Kojima T et al | Shosetsu kyodo hencho hoshasen chiryo [Detailed intensity-modulated radiation therapy] | Chugai Igakusha | 2010 |  |
| 23 | Sano N, Sasaki J, Nishio T et al | Shosetsu taikanbu teii hoshasen chiryo: guideline no shosai to shosha manual [Detailed SBRT: guidelines and irradiation manual] | Chugai Igakusha | 2006 |  |
| 24 | Yamashita H | Hoshasen chiryo case study [Radiotherapy case studies] | Shinko Igaku Shuppansha | 2014 |  |
| 25 | Isobe T, Sato E | Hoshasen chiryo kiso chishiki zukkai note. 2nd ed. [Radiotherapy: basic knowledge illustrated note, 2nd ed.] | Kanehara Shuppan | 2021 |  |
| 26 | Okumura M, Oguchi H, Hoshina M | Hoshasen chiryo gijutsu hyojun text [Standard textbook of radiotherapy technology] | Igaku-Shoin | 2019 |  |
| 27 | Arimura H, Morooka K | Hoshasen chiryo AI to geka chiryo AI [AI for radiotherapy and AI for surgical therapy] | Ohmsha | 2020 |  |
| 28 | Arimura H, Kadoya N | Rediomikusu nyumon [Introduction to radiomics] | Ohmsha | 2021 |  |
| 29 | Karasawa K, Nishio T, Ozawa S | Suisui wakaru hoshasen chiryo butsuri-gaku [An easy guide to radiotherapy physics] | Gakken Medical Shujunsha | 2021 |  |
| 30 | Japan Network for Research and Information on Medical Exposure (J-RIME) | Nihon no shindan sanko reberu (2020-nenban) [Japan diagnostic reference levels (2020)] |  |  | [https://j-rime.qst.go.jp/report/JapanDRL2020_jp.pdf (13 May 2024 accessed)](https://j-rime.qst.go.jp/report/JapanDRL2020_jp.pdf%20(13%20May%202024%20accessed)) |
| 31 | Government of Japan, Ministry of Health, Labour and Welfare | Denri hoshasen shogai boshi kisoku [Regulation on Prevention of Ionizing Radiation Hazards] |  |  | [https://www.japaneselawtranslation.go.jp/](https://www.japaneselawtranslation.go.jp/ja/laws/download/2865/04/s47Coh00410204ja10.0_h27O134.pdf%20%20(13%20May%202024%20accessed))  ja/laws/download/2865/04/ s47Coh00410204ja10.0_h27O134.pdf  (13 May 2024 accessed) |
| 32 | Government of Japan, Ministry of Health, Labour and Welfare | Iryoho shiko kisoku [Enforcement Regulations on the Medical Care Act] |  |  | [https://www.japaneselawtranslation.go.jp/](https://www.japaneselawtranslation.go.jp/ja/laws/download/4007/04/s23Coh00500202ja14.0.pdf%20(13%20May%202024%20accessed)) ja/laws/download/4007/04/ s23Coh00500202ja14.0.pdf  (13 May 2024 accessed) |
